# First report of a novel polymorphism and genetic characteristics of the leporine prion protein (*PRNP*) gene

**DOI:** 10.3389/fvets.2023.1229369

**Published:** 2023-09-22

**Authors:** Dong-Ju Kim, Yong-Chan Kim, Byung-Hoon Jeong

**Affiliations:** ^1^Korea Zoonosis Research Institute, Jeonbuk National University, Iksan, Jeonbuk, Republic of Korea; ^2^Department of Bioactive Material Sciences and Institute for Molecular Biology and Genetics, Jeonbuk National University, Jeonju, Jeonbuk, Republic of Korea; ^3^Department of Biological Sciences, Andong National University, Andong, Republic of Korea

**Keywords:** prion, *PRNP*, rabbit, polymorphism, SNP

## Abstract

Transmissible spongiform encephalopathies (TSEs) have been reported in a broad spectrum of hosts. The genetic polymorphisms and characteristics of the prion protein (*PRNP*) gene have a vital impact on the development of TSEs. Notably, natural TSE infection cases have never been reported in rabbits, and genetic variations of the leporine *PRNP* gene have not been investigated to date. To identify leporine *PRNP* gene polymorphism, we performed amplicon sequencing in 203 rabbits. We report a novel single nucleotide polymorphism on the leporine *PRNP* gene. In addition, we performed a comparative analysis of amino acid sequences of prion protein (PrP) across several hosts using ClustalW2. Furthermore, we evaluated the effect of changes of unique leporine PrP amino acids with those conserved among various species using Swiss-Pdb Viewer. Interestingly, we found seven unique leporine amino acids, and the change of unique leporine amino acids with those conserved among other species, including S175N, Q221K, Q221R, A226Y, A230G, and A230S, was predicted to reduce hydrogen bonds in leporine PrP.

## Introduction

Transmissible spongiform encephalopathies (TSEs), which are infectious and lethal neurodegenerative diseases, manifests as various infections in a wide range of mammalian species, such as scrapie in sheep and goats, chronic wasting disease (CWD) in elk, bovine spongiform encephalopathy (BSE) in cattle, transmissible mink encephalopathy (TME) in mink, feline spongiform encephalopathy (FSE) in cats as well as Creutzfeldt–Jakob disease (CJD), fatal familial insomnia (FFI), Gerstmann-Sträussler Scheinker syndrome (GSS), and kuru in humans (1–9). TSEs are induced by the β-sheet-rich pathogenic isoform of prion protein (PrP^Sc^) originating from a cellular form of prion protein (PrP^C^). In PrP^Sc^ and PrP^C^, ^Sc^ and ^C^ stand for scrapie isoform of the prion protein and cellular prion protein, respectively (10, 11). However, no naturally TSE-infected cases have been reported in rabbits, dogs, and horses. Thus, rabbits, dogs, and horses are considered TSE-resistant animals. Structural stability of PrP has been reported in TSE-resistant animals. In addition, unique amino acids of PrPs in TSE-resistant animals, including S175 in rabbits, S167 in horses, and D163 in dogs, play a major role in PrP stability (12–14). In a circular dichroism (CD) study, rabbit, horse, and dog PrPs showed more stable characteristics against harsh pH conditions compared to those of TSE-susceptible animals, including hamsters and mice (15). In addition, recombinant human PrP with the M209 allele showed increased thermodynamic stability and an inhibitory effect on the conformational conversion of PrP^C^ to PrP^Sc^ (16). In dog PrP, a dog-unique amino acid (D163) contributes to the stability of protein by expanding helix 1 and introducing a negative surface charge (17, 18). In addition, transgenic mice expressing mouse PrP with the D163 allele showed resistance to diverse prion strains, including RML, 301C, 22L, and ME7 (13, 19). In equine PrP, the horse-unique amino acid S167 stabilizes the β2-α2 loop of equine PrP (18, 20). In leporine PrP, the rabbit-unique amino acid S175 has a stronger interaction with N172 than amino acids conserved among other species. The strong side-chain interaction between S175 and N172 forms a helix-capping domain motif that regulates the formation of the β-sheet and is related to an increase in the global stability of leporine PrP (21). Interestingly, the S175N variation disrupts the long-distance interaction of loop 165–172 (22). In addition, rabbits showed resistance to infection by several prion strains, including kuru, CJD, and sheep scrapie (23, 24).

Similar to the major amino acids of TSE-resistant animals mediating resistance to TSEs, *PRNP* gene polymorphisms are vulnerable to mediate TSE infections in sheep, goats, cattle, deer, and humans. In humans, M129V heterozygosity in the *PRNP* gene is rarely observed in variant and sporadic CJD patients (25, 26). In Papua New Guinea highlands, the V127 polymorphism of *PRNP* has been reported as a resistance factor for kuru (27). The key residues of the ovine *PRNP* gene at codons 136, 154, and 171 have been reported as major determinants of susceptibility to scrapie, and the V_136_R_154_Q_171_ allele was considered the most susceptible factor to scrapie (28). Although *PRNP* gene polymorphisms are important factors associated with susceptibility to TSE (12, 29–31) and these polymorphisms have been reported in diverse animals (32–37), *PRNP* gene polymorphisms in rabbits have not been reported to date.

To identify *PRNP* gene polymorphism in rabbits, we performed amplicon sequencing using gene-specific primers. We also performed phylogenetic analysis using Molecular Evolutionary Genetics Analysis (MEGA X Ver. 10.2.0) to estimate evolutionary PrP relationship among 10 species, including deer, mink, cats, mice, cattle, sheep, humans, goats, camels, and rabbits (38). In addition, to identify rabbit-unique amino acids, multiple sequence alignments were performed with amino acid sequences of PrPs from the above-mentioned 10 species. Furthermore, to evaluate the impact of rabbit-unique amino acids on leporine PrP, we evaluated the effect of changes of rabbit-unique amino acids with those conserved among other species on leporine PrP using AMYCO, PolyPhen-2, PROVEAN, and Swiss-Pdbviewer (39–42).

## Methods

### Sample statements

Brain samples from 203 crossbreed rabbits (New Zealand white and Flemish Giant [FG] rabbits) were provided from the Nonghyup slaughterhouse (Korea).

### Genomic DNA extraction

Genomic DNA was isolated from 20 mg of brain tissue from 203 rabbits using Labopass Tissue Genomic DNA Isolation Kit Mini (Cosmogenetech, Seoul, Korea) following the manufacturer's instructions.

### Genetic analysis

Polymerase chain reaction (PCR) was performed using the leporine *PRNP* gene-specific primers: leporine *PRNP*-F (AGACAGGTCCAGGCTGTGAT) and leporine *PRNP*-R (GGA CCAAGAGAGAAGCGAGA). PCR was performed using BioFACT™ *Taq* DNA Polymerase (Biofact, Daejeon, Korea) following the manufacturer's instructions. Amplified PCR products (965 bp) were obtained by electrophoresis on a 1.0% agarose gel and were purified using a Favor Prep Gel/PCR Purification Mini Kit (FAVORGEN, Pingtung County, Taiwan). PCR products were analyzed using an ABI 3730 sequencer (ABI, Foster City, California, USA). Sequencing results were examined by Finch TV software (Geospiza Inc, Seattle, WA, USA).

### Phylogenetic analysis

Phylogenetic analysis according to amino acid sequences of PrPs from 10 species (deer, mink, cats, mice, cattle, sheep, humans, goats, camels, and rabbits) was performed using MEGA X Ver. 10.2.0 (Swiss Institute of Bioinformatics, Geneva, Switzerland) (Supplementary Table S1) (38). The evolutionary tree was constructed by the neighbor-joining method with 3,000 bootstrap tests. The Poisson correction method was used to compute the evolutionary distances, which are reported in units of the number of amino acid changes per site.

### Multiple sequence alignments

Multiple sequence alignments of sequences from 10 species-specific PrPs (deer, mink, cats, mice, cattle, sheep, humans, goats, camels, and rabbits) were conducted using ClustalW2 (43).

### *In silico* analysis of amino acid changes from leporine PrP

The effect on leporine PrP amino acid changes was predicted using three *in silico* programs, PolyPhen-2, PROVEAN, and AMYCO. PolyPhen-2 evaluated the impact on protein function based on numerous features, including the sequence, structure, and phylogenetic information. The predicted effects ranged from benign to possibly damaging or probably damaging. PROVEAN scores were calculated to predict the impact of protein sequence variations on protein function by clustering BLAST hits based on the homologs obtained from the National Center for Biotechnology Information (NCBI) database. AMYCO evaluated the impact on the aggregation propensity of proteins using pWALTZ and PAPA algorithms. An AMYCO score less than 0.45 indicates low amyloid property.

### Three-dimensional structural analysis of leporine PrP

The nuclear magnetic resonance (NMR) of leporine PrP (protein data bank [PDB] ID: 2FJ3) was visualized using Swiss-PdbViewer 4.1. The structural change resulting from the amino acid changes was analyzed using Swiss-PdbViewer 4.1. Hydrogen bonds were predicted when a hydrogen atom fell within the range of 1.2 to 2.76 Å from a “compatible” donor atom.

### Statistical analysis

The Hardy-Weinberg equilibrium (HWE) test was estimated using Haploview version 4.2 (Broad Institute, Cambridge, MA, USA).

## Results

### Identification of a novel SNP in the leporine *PRNP* gene in 203 rabbits

**Figure 1:**
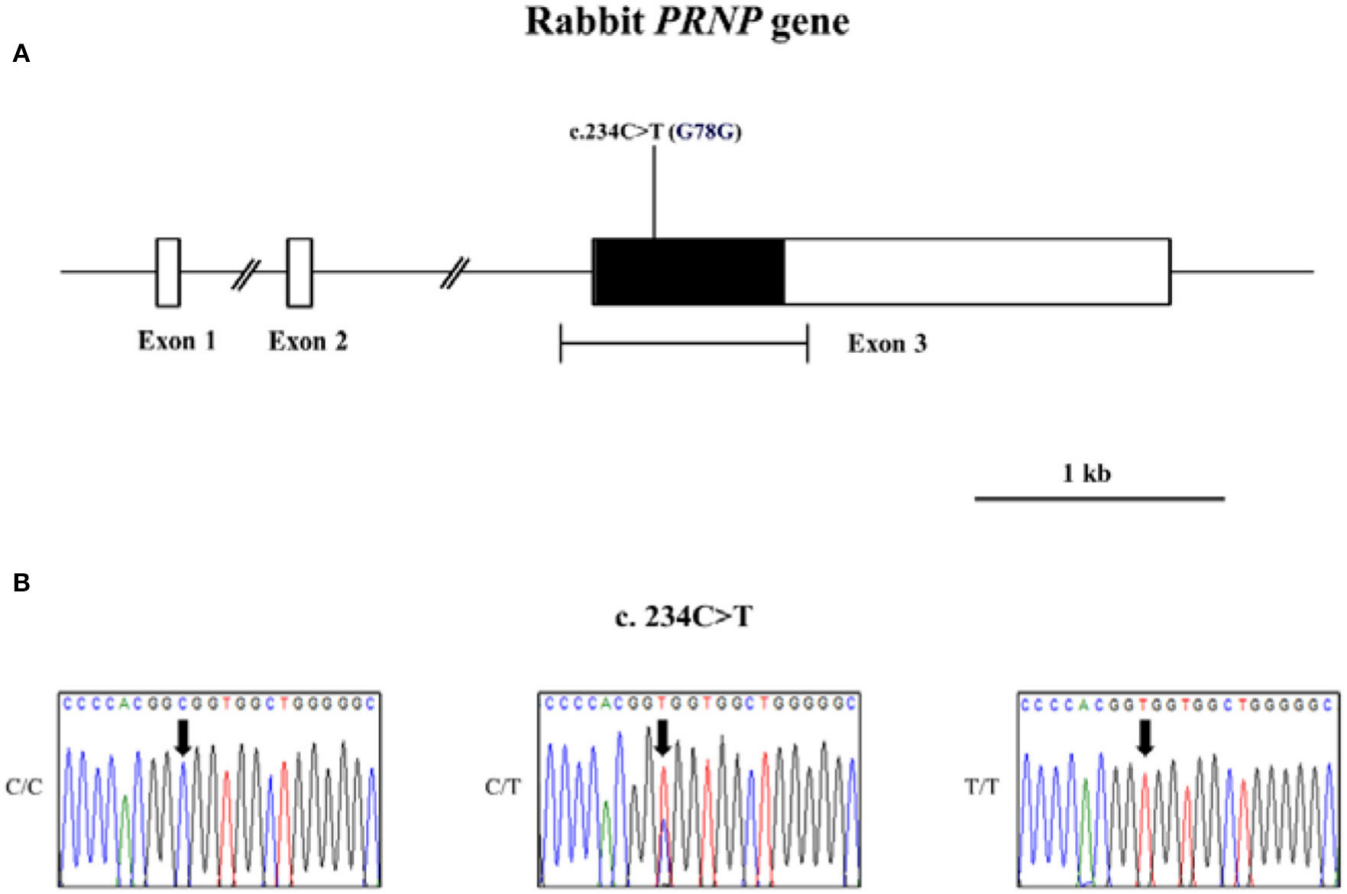
Gene map and electropherograms of a novel single nucleotide polymorphism (SNP) identified in the leporine prion protein (*PRNP*) gene on chromosome 4. **(A)** Map and a novel polymorphism identified in the leporine *PRNP* gene. The open reading frame (ORF) is visually represented by a shaded block, and the 5′ and 3′ untranslated regions (UTRs) are depicted by white blocks. The horizontal bar with edges indicates the regions sequenced. **(B)** Electropherograms of the novel SNP c.234C>T (p.G78G). Electropherograms showing the three genotypes at codon 78. Left panel: GGC/GGC; middle panel: GGT/GGC; right panel: GGT/GGT. Gene map and novel polymorphism identified in the leporine *PRNP* gene. The ORF is indicated by a shaded block, and the 5′ and 3′ UTRs are indicated by white blocks. The horizontal bar with edges indicates the regions sequenced. **(B)** Electropherograms of the novel SNP c.234C>T (p.G78G). Electropherograms showing the three genotypes at codon 78. Left panel: GGC/GGC; middle panel: GGT/GGC; right panel: GGT/GGT.

The leporine *PRNP* gene is composed of three exons on chromosome 4. We performed PCR amplification to target the open reading frame (ORF) of the leporine *PRNP* gene, which is located in exon 3, using gene-specific primers. The length of the amplicon was 965 bp, and the sequence was identical to that reported in GenBank (Gene ID: 100008658). To investigate genetic variations in the leporine *PRNP* gene, we performed PCR with genomic DNA from 203 rabbits. Notably, we identified only one novel synonymous SNP (c.234C>T, p.G78G) in the ORF region (Figure 1, Supplementary Figure S1). Of 203 rabbits, 181 (89.2%) were homozygous for the C allele, 1 (0.4%) was homozygous for the T allele, and 21 (10.4%) were heterozygous (10.4%) at codon 78. The allele frequency of c.234C>T was 383 C alleles (94.3%) and 23 T alleles (5.7%) in 203 rabbits. This SNP was in HWE (Table 1).

**Table 1:** Genotype and allele frequencies of the *PRNP* polymorphism in rabbits.

**Polymorphism**		**Total, *n***	**Genotype frequency**, ***n*** **(%)**			**Allele frequency**, ***n*** **(%)**		**HWE**
c.234C>T	p.G78G	203	C/C 181 (89.2)	C/T 21 (10.4)	T/T 1 (0.4)	C 383 (94.3)	T 23 (5.7)	0.647

HWE, Hardy-Weinberg Equilibrium.

### Phylogenetic analysis among 10 species

**Figure 2:**
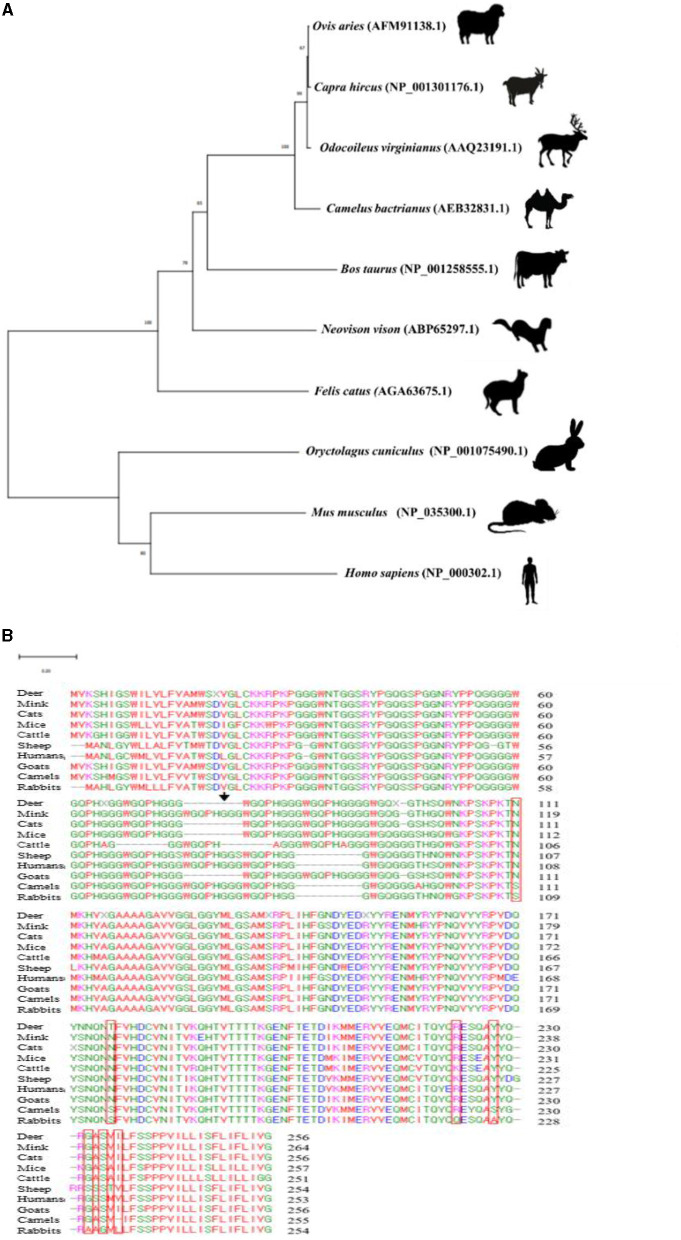
Phylogenetic analysis and multiple sequence alignments of the amino acid sequences of the PrPs in 10 species. **(A)** Phylogenetic analysis included amino acid sequences of deer (*Odocoileus virginianus*, AAQ23191.1), cattle (*Bos taurus*, NP_001258555.1), sheep (*Ovis aries*, AFM91138.1), mink (*Neovison vison*, ABP65297.1), cats (*Felis catus*, AGA63675.1), rabbits (*Oryctolagus cuniculus*, NP_001075490.1), mice (*Mus musculus*, NP_035300.1), goats (*Capra hircus*, NP_001301176.1), camels (*Camelus bactrianus*, AEB32831.1), and humans (*Homo sapiens*, NP_000302.1). Evolutionary analysis was carried out using Molecular Evolutionary Genetics Analysis (MEGA) X software based on the neighbor-joining method. The branch lengths indicating evolutionary distances were computed by using the minimum evolution method. The numbers displayed at the branches represent the percentage of bootstrap replicates in which the associated taxa formed a cluster together in the bootstrap test (3,000 replicates). The scale bar represents a length of 0.20. **(B)** The PrP sequences of deer (*Odocoileus virginianus*, AAQ23191.1), mink (*Neovison vison*, ABP65297.1), cats (*Felis catus*, AGA63675.1), mice (*Mus musculus*, NP_035300.1), cattle (*Bos taurus*, NP_001258555.1), sheep (*Ovis aries*, AFM91138.1), humans (*Homo sapiens*, NP_000302.1), goats (*Capra hircus*, NP_001301176.1), camels (*Camelus bactrianus*, AEB32831.1), and rabbits (*Oryctolagus cuniculus*, NP_001075490.1) were aligned with ClustalW2. The red box indicates rabbit-unique amino acids. The black arrow indicates a novel SNP found in this study.

Phylogenetic analysis was performed to estimate the evolutionary relationship among the amino acid sequences of PrPs in 10 species (deer, mink, cats, mice, cattle, sheep, humans, goats, camels, and rabbits). The rabbit showed a closer evolutionary relationship with mice and human than deer, mink, cats, cattle, sheep, goats, and camels. The subclades of cattle, sheep, goats, camels, and deer, which are known as artiodactyla, were clustered with the farthest evolutionary distances from rabbits (Figure 2A).

### Identification of rabbit-unique amino acids using multiple sequence alignments

To identify rabbit-unique amino acids, we aligned the amino acid sequences of PrPs of mink, deer, cats, mice, cattle, sheep, humans, goats, camels, and rabbits (Supplementary Table S1). The amino acid residue numbering was determined by reference sequence of the leporine PrP. A total of seven rabbit-unique amino acids, including S109, S175, Q221, A226, A230, G232, and L234 were found. In addition, interspecies-specific conserved amino acids were found at 11 residues (N109, N175, K221, R221, Y226, F226, G230, S230, S232, I234, and V234) (Figure 2B).

### Estimation of the influence on rabbit-unique amino acids on leporine PrP

To investigate the influence on rabbit-unique amino acids, we estimated the effect of amino acid changes from rabbit-unique amino acids into interspecies-specific conserved amino acids (Figure 2B) on leporine PrP using PolyPhen-2, PROVEAN, and AMYCO (Supplementary Table S2). The amyloid propensity of wild-type leporine PrP was predicted to have a score of 0.27 using AMYCO. Notably, except for S175T (0.23), all amino acid changes, including S109N, S175N, Q221K, Q221R, A226Y, A230G, A230S, G232S, L234I, and L234V, were predicted to have a score of 0.27 using AMYCO (Supplementary Table S2). AMTCO scores <0.45 and >0.45 denote low and high aggregation propensity, respectively. In addition, the effects of all amino acid changes on leporine PrP were evaluated to be “Benign” and “Neutral” using PolyPhen-2 and PROVEAN, respectively (Supplementary Table S2).

**Figure 3:**
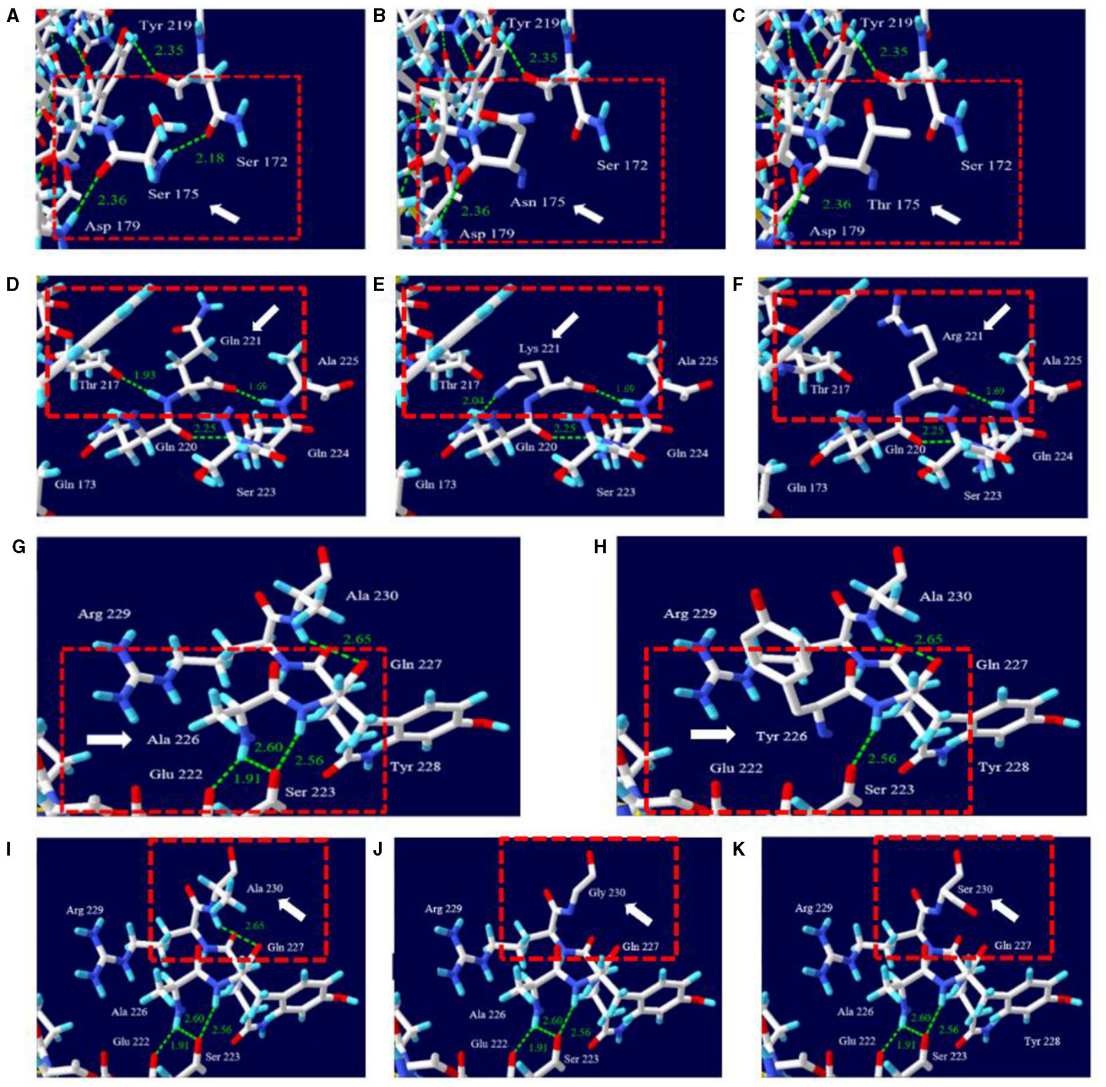
Prediction of the 3D structure and hydrogen bonds of PrP according to amino acid changes of rabbit-unique amino acids with interspecies-specific conserved amino acids. **(A)** 3D structure of leporine PrP carrying the S175 allele, **(B)** 3D structure of leporine PrP carrying the N175 allele, **(C)** 3D structure of leporine PrP carrying the T175 allele, **(D)** 3D structure of leporine PrP carrying the Q221 allele, **(E)** 3D structure of leporine PrP carrying the K221 allele, **(F)** 3D structure of leporine PrP carrying the R221 allele, **(G)** 3D structure of leporine PrP carrying the A226 allele, **(H)** 3D structure of leporine PrP carrying the Y226 allele, **(I)** 3D structure of leporine PrP carrying the A230 allele, **(J)** 3D structure of leporine PrP carrying the G230 allele, and **(K)** 3D structure of leporine PrP carrying the S230 allele. The target amino acid residues are indicated by the white arrow. The adjacent amino acid residues are highlighted in the red box indicates adjacent amino acid residues. The presence of hydrogen bonds is represented by green dotted lines. The distance of the hydrogen bonds is indicated by the green numbers.

The effects of changes of rabbit-unique amino acids with interspecies-unique conserved amino acids on the 3D protein structure and hydrogen bonds of leporine PrP were analyzed using the Swiss-Pdb Viewer (Figure 3). S175 was expected to have hydrogen bonds with D179 (2.36 Å) and with S172 (2.18 Å), respectively (Figure 3A). However, N175 expected to form a hydrogen bond (2.36 Å) with D179 (Figure 3B). T175 was also expected to form a hydrogen bond with D179 (2.36 Å) (Figure 3C). Q221 was expected to form a with T217 (1.93 Å) and with A225 (1.69 Å), respectively (Figure 3D). K221 was expected to have one hydrogen bond with Q220 (2.04 Å) and with A225 (1.69 Å), respectively (Figure 3E). R221 was expected to form a hydrogen bond with A225 (1.69 Å) (Figure 3F). A226 was expected to form two hydrogen bonds with E222 and S223 (1.91 and 2.60 Å) (Figure 3G). Y226 was expected to form no hydrogen bonds (Figure 3H). A230 was expected to have single E227 (2.65 Å) (Figure 3I). G230 and S230 were predicted to have no hydrogen bonds (Figures 3J, K).

## Discussion

To identify susceptibility/resistance factors for prion diseases, the structure of PrP was analyzed using NMR, molecular dynamics (MD), and X-ray crystallography in previous studies (18, 44, 45). In rabbits, a TSE-resistant animal, PrP has a helix-capping domain, a highly ordered β2–α2 loop and stable salt bridges and exhibits stable characteristics that may contribute to resistance to TSE infection. However, leporine *PRNP* polymorphism affecting protein structure has not been reported in previous studies. Interestingly, we found only one novel synonymous SNP located on the tandem repeat region of the leporine PrP that does not affect the amino acid sequences and structure of leporine PrP (Figures 1A, B). To the best of our knowledge, an association between synonymous SNPs and susceptibility to prion diseases has not been reported thus far. Notably, among TSE-resistant animals, SNPs have not been reported in chicken *PRNP*. In addition, a nonsynonymous SNP has been reported in that of horse *PRNP*, but this SNP did not affect the stability of the horse-unique PrP structure (12, 46, 47). Compared to TSE-susceptible animals, which have several *PRNP* gene polymorphisms, TSE-resistant animals have a small number of polymorphisms. Based on these studies, the number of polymorphisms could be associated with resistance to TSEs.

Synonymous SNPs may modulate translation speed and protein stability (48, 49). Since prion diseases are associated with protein stability, further validation of the relationship between the synonymous SNP of the leporine *PRNP* gene and the structural stability of leporine PrP at the molecular level is highly desirable. In the present study, a polymorphism was identified within the ORF of the leporine *PRNP* gene in exon 3. However, it is possible that other influencing genetic polymorphisms exist in non-ORF regions. Therefore, further investigation in the non-ORF regions of the leporine *PRNP* gene is needed in the future.

Some evidence regarding prion resistance in rabbits exists. Firstly, no naturally occurring cases of TSE infection have been reported in rabbits. Secondly, *in vitro* screening has identified key amino acids related to the misfolding of leporine PrP (50). However, transgenic mice overexpressing leporine PrP and transgenic rabbits carrying ovine PrP showed vulnerability to prion infection (51, 52). However, since the path mechanisms of prion diseases are influenced by various factors, including the PrP sequence, cofactors, and infection route, etc., studies using transgenic animals may not accurately represent the natural prion disease-resistance observed in rabbits. Consequently, there is a strong need for further investigation using a direct infection model using rabbits.

To identify an additional prion resistance factor of leporine PrP, we found seven rabbit-unique amino acids (Figure 2B). Unexpectedly, deleterious effects based on changes of rabbit-unique amino acids with interspecies-specific conserved amino acids have not been estimated by PolyPhen-2, PROVEAN, and AMYCO (Supplementary Table S2). Given that PolyPhen-2, PROVEAN, and AMYCO programs evaluate the alteration of protein function based on homologs collected from databases, stability-related properties may haven't been predicted by the programs (39, 40, 42, 53). Our results showed that PolyPhen-2, PROVEAN, and AMYCO haven't been predicted to have deleterious effects according to the changes of rabbit-unique amino acids. However, since the rabbit-unique amino acids may affect the TSE resistance, we evaluated the amino acid changes effect of rabbit-unique amino acids by 3D structure analysis (Figure 3).

Among seven rabbit-unique amino acids, S175 has been previously reported to be a crucial factor in TSE resistance that confers stability and expansion of helix 2 (21, 22). We also observed that leporine PrP with the N175 allele has fewer hydrogen bonds (Figure 3), which are essential to the secondary structure and stability of proteins (54–56). In addition to S175, rabbit-unique amino acids, including Q221, A226, and A230, were observed, and these amino acids play a crucial role in the formation of hydrogen bonds (Figure 3). Given that one hydrogen bond provides protein stabilization (1.0 kcal/mol), further studies are needed to investigate the impact of rabbit-unique amino acids on leporine PrP. To determine the effect of rabbit-unique amino acids on the stability of leporine PrP, further analysis using CD, NMR, and X-ray crystallography should be performed in the future. Furthermore, TSE infection in transgenic rabbit using leporine PrP carrying interspecies-specific conserved amino acids instead of rabbit-unique amino acids should be performed to evaluate susceptibility of unique amino acids to prion diseases.

## Conclusions

In summary, we identified one novel synonymous polymorphism of the leporine *PRNP* gene. In addition, leporine PrP has seven rabbit-unique amino acids compared with nine species including deer, mink, cats, mice, cattle, sheep, humans, goats, and camels. Hydrogen bonds of leporine PrP were decreased by the changes of rabbit-unique amino-acids into amino acids conserved among species, including S175, Q221, A226, and A230.

## Data availability statement

The data presented in the study are deposited in the DRYAD repository (https://datadryad.org/stash/share/kG8H4tE-LwkAk6VQcdQcuX3vlfbmem5DlKxqZNMgdWU).

## Ethics statement

The animal study was approved by Institute of Animal Care and Use Committee of Jeonbuk National University. The study was conducted in accordance with the local legislation and institutional requirements.

## Author contributions

D-JK, YC-K, and B-HJ conceived and designed the experiment, analyzed the data, and wrote the paper. D-JK and Y-CK performed the experiments. All authors read and approved the final manuscript.
