# Supplementary material for: First report of a novel polymorphism and genetic characteristics of the leporine prion protein (PRNP) gene
Source: Front Vet Sci. 2023 Sep 22;10:1229369. doi: 10.3389/fvets.2023.1229369 (PMC10556520; doi:10.3389/fvets.2023.1229369)
Supplement: Supplementary file 1 [file Data_Sheet_1.docx]

**Supplementary Table S1.** National Center for Biotechnology Information (NCBI) GenBank IDs of the amino acid sequences of the 10 species analyzed in this study.

| Common name | Scientific name | GenBank ID |
| --- | --- | --- |
| Deer | *Odocoileus virginianus* | AAQ23191.1 |
| Mink | *Neovison vison* | ABP65297.1 |
| Cat | *Felis catus* | AGA63675.1 |
| Mouse | *Mus musculus* | NP_035300.1 |
| Cattle | *Bos taurus* | NP_001258555.1 |
| Sheep | *Ovis aries* | AFM91138.1 |
| Human | *Homo sapiens* | NP_000302.1 |
| Goat | *Capra hircus* | NP_001301176.1 |
| Camel | *Camelus bactrianus* | AEB32831.1 |
| Rabbit | *Oryctolagus cuniculus* | NP_001075490.1 |

**Supplementary** Table S2. *In silico* evaluation of the effect according to substitutions from rabbit-unique amino acids to interspecific conserved amino acids.

| Substitution | AMYCO |  | PolyPhen-2 | |  | PROVEAN | |  |
| --- | --- | --- | --- | --- | --- | --- | --- | --- |
|  | Score |  | Score | Prediction |  | Score | Prediction |  |
| WT | 0.27 |  | - | - |  | - | - |  |
| S109N | 0.27 |  | 0 | Benign |  | 1.023 | Neutral |  |
| S175N | 0.27 |  | 0 | Benign |  | 0.415 | Neutral |  |
| S175T | 0.23 |  | 0 | Benign |  | 0.154 | Neutral |  |
| Q221K | 0.27 |  | 0 | Benign |  | 0.452 | Neutral |  |
| Q221R | 0.27 |  | 0 | Benign |  | 0.232 | Neutral |  |
| A226Y | 0.27 |  | 0.106 | Benign |  | -0.098 | Neutral |  |
| A230G | 0.27 |  | 0 | Benign |  | 0.512 | Neutral |  |
| A230S | 0.27 |  | 0.002 | Benign |  | 0.267 | Neutral |  |
| G232S | 0.27 |  | 0.001 | Benign |  | -0.090 | Neutral |  |
| L234I | 0.27 |  | 0 | Benign |  | 0.267 | Neutral |  |
| L234V | 0.27 |  | 0 | Benign |  | 0.397 | Neutral |  |


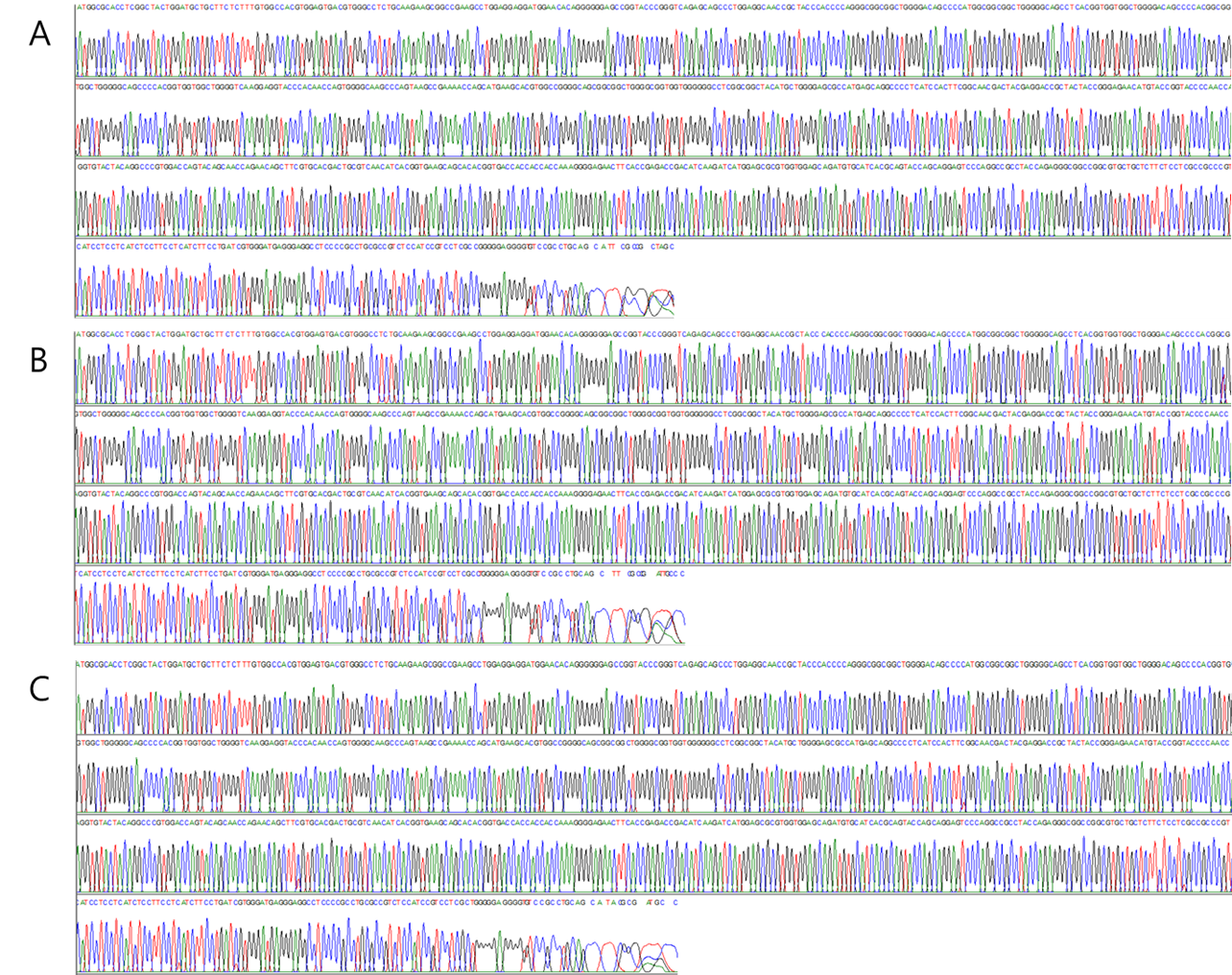


**Supplementary Figure S1.** Sanger sequencing data of the SNP of leporine *PRNP* gene identified in this study. (A) GGC/GGC, (B) GGT/GGC, (C) GGT/GGT.
